# Urban greening with shrubs can supercharge invertebrate abundance and diversity

**DOI:** 10.1038/s41598-024-58909-8

**Published:** 2024-04-16

**Authors:** Mahmuda Sharmin, Mark G. Tjoelker, Manuel Esperon-Rodriguez, Alihan Katlav, Amy-Marie Gilpin, Paul D. Rymer, Sally A. Power

**Affiliations:** 1 Hawkesbury Institute for the Environment, Western Sydney University, Locked Bag 1797, Penrith, NSW 2751 Australia; 2 Department of Forestry and Environmental Science, Shahjalal University of Science and Technology, Sylhet, 3114 Bangladesh; 3 School of Science, Western Sydney University, Locked Bag 1797, Penrith, NSW 2751 Australia

**Keywords:** Urban ecosystems, Functional diversity, Vegetation management, Mid-story vegetation, Young trees, Invertebrate abundance and richness, Ecology, Ecology

## Abstract

In urban areas, diverse and complex habitats for biodiversity are often lacking. This lack of diversity not only compromises essential ecological processes, such as pollination and nutrient cycling, but also diminishes the resilience of urban ecosystems to pests and diseases. To enhance urban biodiversity, a possible solution is to integrate shrubs alongside trees, thereby increasing the overall amount of vegetation, structural complexity and the associated resource diversity. Here, using a common garden experiment involving a variety of trees and shrubs planted alone and in combination, we evaluate how canopy-associated invertebrate assemblages are influenced by vegetation type. In particular, we test whether the presence of shrubs, alone or with trees, results in increased abundance and taxonomic richness of invertebrates, compared to trees on their own. We found that the overall abundance of invertebrates, and that of specific functional groups (e.g., herbivores, pollinators, detritivores), was higher on shrubs, compared to trees, and when trees and shrubs were planted in combination (relative to trees on their own). Our results suggest that planting shrub and tree species with wide and dense crowns can increase the associated abundance and taxonomic and functional group richness of invertebrate communities. Overall, our findings indicate that urban planning would benefit from incorporating shrubs alongside urban trees to maximise invertebrate abundance, diversity and function in urban landscapes.

## Introduction

Urbanization has negatively impacted ecological functioning through habitat fragmentation and biodiversity loss^1,2^. However, establishment of sustainable greenspaces in urban settings can improve ecosystem services and the quality of life in cities^3^. A robust correlation exits between the diversity of invertebrates and provision of ecosystem services such as pollination for food production, pest biocontrol and nutrient turnover^4–9^. Invertebrates require access to a wide range of resources, such as food, shelter and sites for reproduction. However, urbanization has resulted in substantial changes in land use, reducing the structural diversity and extent of available habitat, leading to associated declines in the diversity and abundance of many invertebrate species in urban areas^10^. Developing strategies that incorporate biodiversity into urban areas must be a priority to promote and preserve healthy and functional urban environments^11,12^.

In most urban areas, a significant proportion of green space consists of isolated trees that have distinct, separate crowns^13^. These trees are typically maintained to an aesthetic standard that is often associated with relatively low levels of habitat complexity^14,15^. This is notwithstanding the fact that habitats with more complex vegetation structure (e.g., multi-layered canopies such as trees growing alongside shrubs and grasses^16^) can potentially harbour high invertebrate species abundance and richness^17–19^. Indeed, remnant forests, golf courses, parks and gardens can provide structurally complex vegetation of high volume and species richness, and have been associated with high levels of invertebrate biodiversity^20,21^, although they typically occupy only a small proportion of urban green areas^22^. The presence of mid-storey shrub vegetation can play a crucial role in establishing a corridor between trees and ground-level vegetation and soil, facilitating the movement of animals and invertebrates across the urban matrix^23^. Moreover, shrubs can improve nutrient cycling by providing abundant leaf litter^24^.

In recent years, there has been a surge in scientific investigations targeting invertebrate populations within urban ecosystems^25–28^. This surge reflects growing recognition of the ecological significance of invertebrates in urban settings, driven by concerns about biodiversity conservation, urbanization effects and the role of invertebrates in sustaining essential ecosystem services. In general, most urban biodiversity studies have focused on only a small sub-set of invertebrates (e.g., pollinators, ants, ground dwelling beetles)^25,29–31^, despite there being many functional groups and diverse invertebrate taxa that play important roles in ecosystem functioning. While the impact of vegetation structural complexity on invertebrate communities has been the topic of a number of studies^17,22,32^, the extent of invertebrate associations specifically with mid-story vegetation (e.g., shrubs) remains relatively under-explored. Furthermore, invertebrate diversity varies temporally across seasons often in relation to plant growth and phenology, invertebrate activity, changing temperature and invertebrate life history stages^33^. The lack of urban studies covering multiple invertebrate taxa and functional groups across seasons limits our ability to plan future cities that effectively foster biodiversity. This study comprised a common garden experiment with young trees and shrubs that are commonly recommended and planted in street-scapes in New South Wales (NSW), Australia. We hypothesised that (1) shrubs can support a greater abundance and richness of invertebrates than young trees, and that (2) they therefore increase local-scale biodiversity when planted alongside trees. These hypotheses are grounded on the assumption that shrubs differ in their morphology relative to trees, reflecting their typically shorter stature and multiple stems arising at or near the surface of the ground (with its associated fauna), allowing them to harbour a more abundant and diverse invertebrate community. The implications of our findings are relevant for planning and management of greener, more biodiverse and ecologically sustainable urban environments.

## Results

### Invertebrate abundance and richness are greater in shrubs than trees

Over the course of the austral summer 2019/20, spring 2020 and summer 2020/21, we used the branch beating sampling method to collect invertebrates in the canopies of shrubs and young trees. Invertebrates were classified into seven functional groups: detritivores, herbivores, parasitoids, pollinators, predators, sap suckers and scavengers (Supplementary Table S1). Our analysis assessed the taxonomic richness and abundance of invertebrates present, as well as the abundance of different invertebrate functional groups, for individual plants of each of four tree and four shrub species and for contrasting vegetation type treatments (i.e., ‘shrub only’, ‘tree only’, ‘tree and shrub’). Before pooling data for individual tree and shrub groups across the different planting combinations, we tested whether invertebrate abundance and richness differed for trees and shrubs when grown alone *vs.* when grown with each other (i.e., tree only vs. tree growing with shrubs; shrub only vs shrubs growing with a tree). We found no significant difference in invertebrate abundance or richness between shrubs growing on their own or alongside a tree (abundance, t = 0.89, *p* = 0.37; richness, t = 0.40, *p* = 0.69), nor was there a significant difference between trees growing on their own or those growing adjacent to shrubs (abundance, t = 0.21, *p* = 0.84; richness, t = 0.68, *p* = 0.50) (Supplementary Fig. S1). Therefore, subsequent tree *versus* shrub comparisons included individuals growing both on their own and in combination with the contrasting plant functional type.

**Figure 1:**
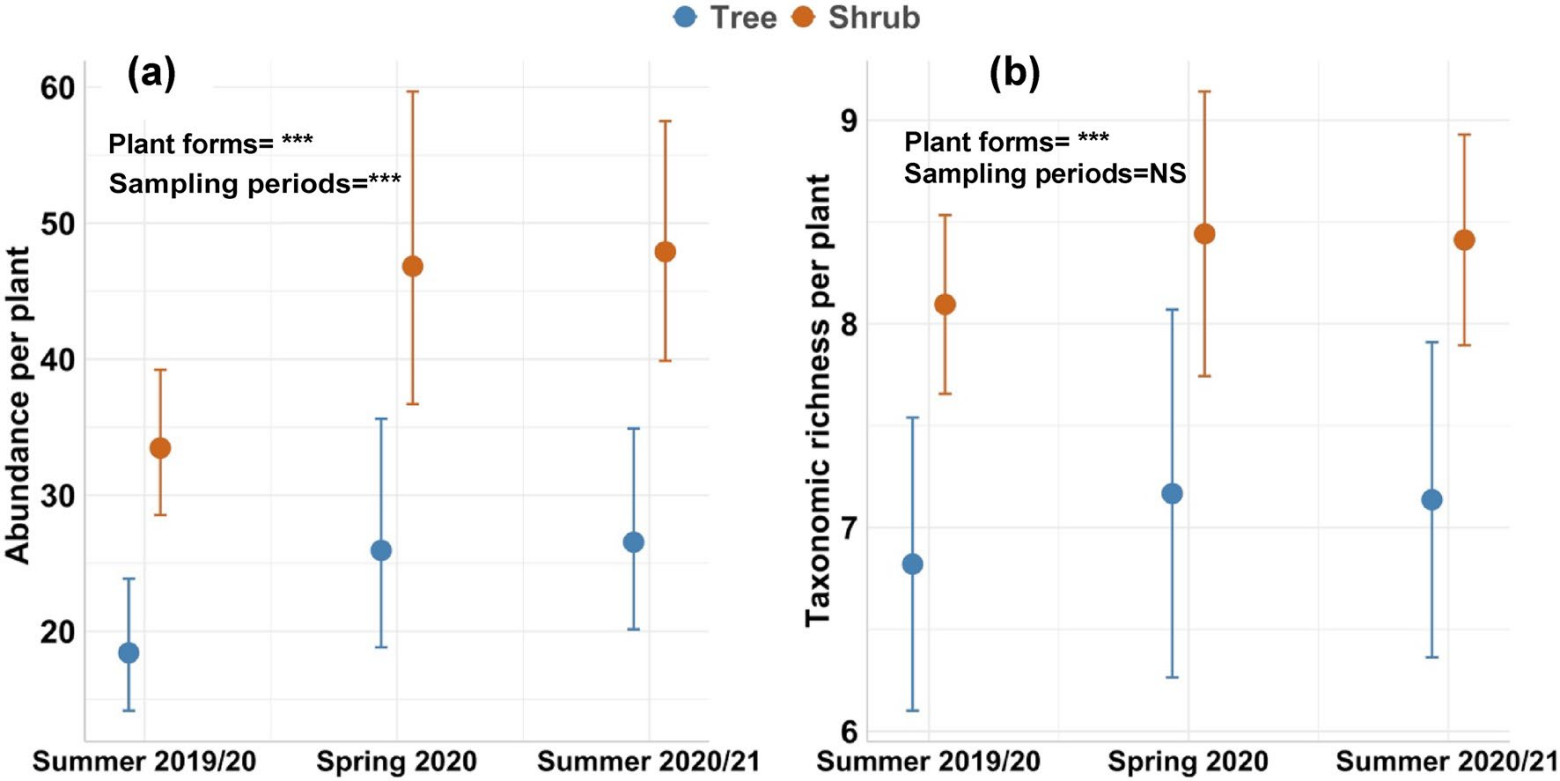
Pairwise comparisons of abundance (**a**) and taxonomic richness (**b**) of invertebrates on individual plants of shrubs and trees (pooled across the tree only, shrub only and tree and shrub vegetation treatments) for three sampling periods—austral summer 2019/20, spring 2020 and summer 2020/21. Data shown are for individual plants pooled across the four tree or shrub species. Circles and error bars depict mean abundance and standard errors (*n* = 32 for trees; *n* = 128 for shrubs). Supplementary Table S2 summarises ANOVA results of the *lmer* model for each figure. Plot ID and species were included as random factors in models. Asterisks represent statistical significance: ***P < 0.001.

Invertebrate abundance differed significantly between shrubs and trees (i.e., *X*^2^ = 19.83; *p* = 0.01) (Fig. 1a); Supplementary Table S2). Abundance varied from 0 to 979 individuals on individual shrubs (mean 95.72 ± 4.53 standard error) and from 0 to 362 individuals (53.85 ± 5.05) on individual trees. Invertebrate taxonomic richness also differed significantly between shrubs and trees (*X*^2^ = 12.50; *p* = 0.01), ranging from 0 to 19 taxa for shrubs (7 ± 0.28) and 0 to 16 taxa for trees (9.26 ± 0.23) across all sampling periods (Fig. 1b).

### Invertebrate abundance and richness are higher in association with shrubs

**Figure 2:**
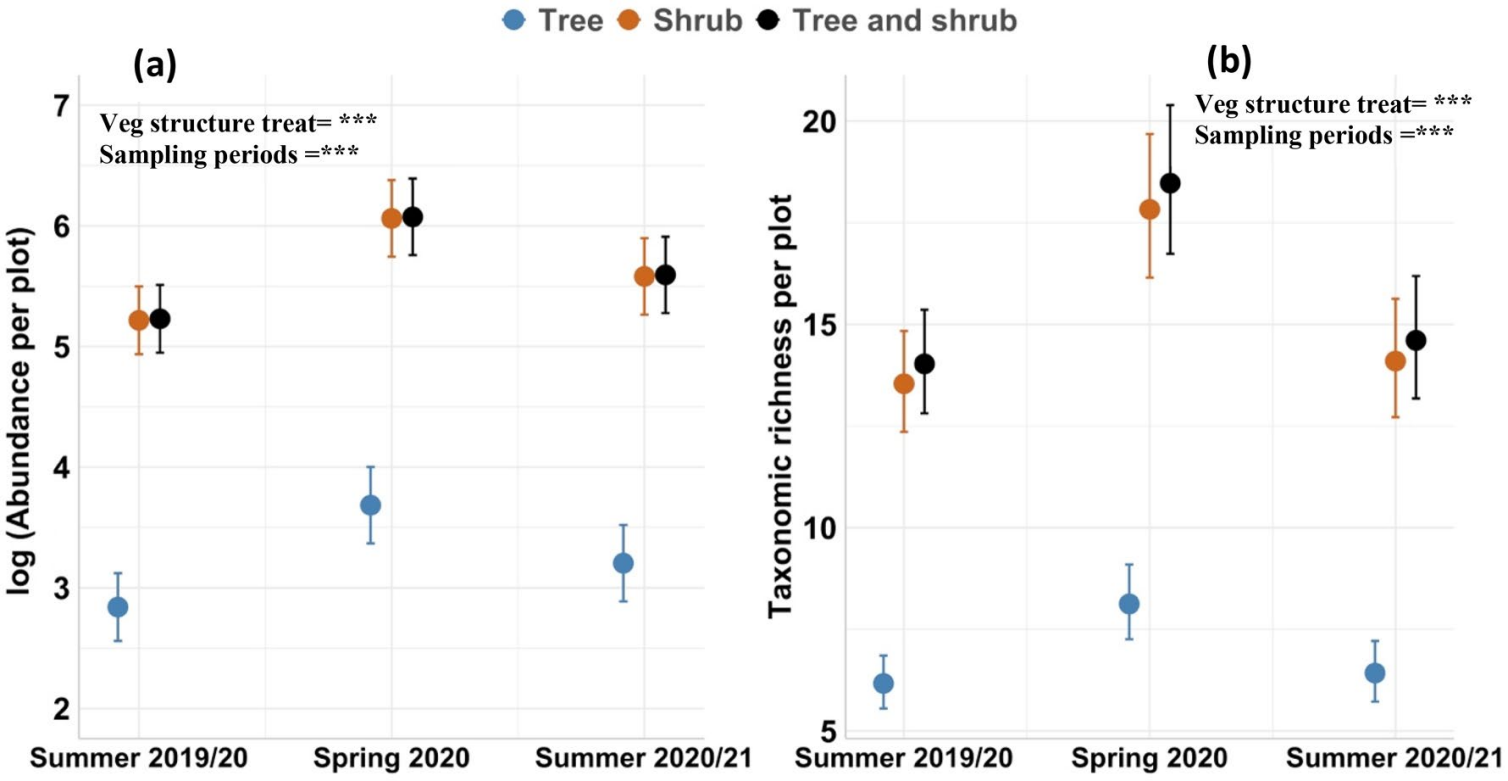
Pairwise comparisons of the abundance (**a**) and taxonomic richness (**b**) of invertebrates at the plot-level among vegetation type treatments (i.e., ‘tree only’, ‘shrub only’ and ‘tree and shrub’ treatments) across sampling periods. Data shown are at the plot-scale (4 m^2^). Circles and error bars depict means and standard errors (*n* = 16 plots). Abundance data are shown on a natural log scale. Supplementary Table S2 summarises ANOVA results of the *lmer* model for each figure. Plot ID was included as a random effect. Asterisks represent statistical significance: ***P < 0.001.

Invertebrate abundance and richness differed among the vegetation type treatments and across sampling periods (Fig. 2). Both ‘shrub only’ and ‘tree and shrub’ treatments were associated with significantly higher numbers of invertebrates than the ‘tree only’ treatment (Fig. 2a; Supplementary Tables S2 and S3). Invertebrate richness varied up to two-fold among vegetation structure treatments across sampling periods (Fig. 2b). Both ‘shrub only’ and ‘tree and shrub’ plots were also associated with higher invertebrate richness than tree-only plots; taxonomic richness varied significantly (*X*^2^ = 214.20; *p* = 0.01) across all sampling periods, with greater richness observed in spring compared to the summer sampling periods (Fig. 2b).

### Plant traits and vegetation structure affect invertebrate abundance and taxonomic richness

We used linear mixed-effects models (*lmer*) to assess the effect of traits of individual shrubs or trees and vegetation structure treatments (i.e., ‘shrub only’, ‘tree only’, ‘tree and shrub’) on the abundance and richness of invertebrates. Plot ID and sampling periods were included as random effects in the models. For individual shrubs pooled across all vegetation structure treatments, invertebrate abundance and taxonomic richness were significantly positively associated with canopy volume, leaf area (LA) and the presence of flowers. For individual trees, there were only strong associations of invertebrate abundance with the presence of flowers, and of invertebrate richness with LA (Table 1).

**Table 1:** ANOVA results of the three *lmer* models for individual trees and shrubs for each of the explanatory variables, canopy volume, leaf area (LA) per plant and presence of flowers, for invertebrate abundance and taxonomic richness data.

Response variable	Explanatory variables	Abundance (ln)			Richness (sqrt)	
		df	*X*^2^	*p* value	*X*^2^	*p* value
Shrubs (n = 128)	Volume	1	116.0	0.001	138.3	0.001
	LA	1	4.06	0.04	6.92	0.01
	Presence of flowers	1	48.5	0.001	49.4	0.001
Trees (n = 32)	Volume	1	0.26	ns	0.38	ns
	LA	1	0.09	ns	7.44	0.01
	Presence of flowers	1	16.0	0.001	2.08	ns

Data shown for individual trees or shrubs are pooled across all species and planting combinations. Plot ID and sampling periods were included as random effects in the models.

Invertebrate abundance was positively related with total plot-level canopy volume and the number of flowering plants in both ‘shrub only’ and ‘tree and shrub’ treatments (Table 2). In the ‘tree only’ treatment, however, invertebrate abundance and richness were only positively related with the presence of flowers. In the ‘shrub only’ treatment, invertebrate richness was positively related with canopy volume, whereas in the ‘tree and shrub treatment’, richness was correlated with both canopy volume and LA.

**Table 2:** Results of four *lmer* models for each explanatory variable (total canopy volume, total leaf area per plant (LA) and number of flowering plants), for invertebrate abundance and taxonomic richness among vegetation type treatments (tree only, shrub only, tree and shrub).

Vegetation structure treatments	Explanatory variables	Abundance (ln)			Richness (ln)	
		df	*X*^2^	*p* value	*X*^2^	*p* value
Tree only treatment (n = 16)	Total volume	1	0.11	ns	1.11	ns
	Total LA	1	0.03	ns	1.18	ns
	No. of flowering plants	1	25.85	0.001	20.48	0.001
Shrub only treatment (n = 16)	Total volume	1	174.62	0.001	150.28	0.001
	Total LA	1	2.98	ns	2.52	ns
	No. of flowering plants	3	23.61	0.001	2.51	ns
Tree and shrub treatment (n = 16)	Total volume	1	18.84	0.001	18.01	0.001
	Total LA	1	6.6	0.01	9.02	0.002
	No. of flowering plants	4	4.20	0.04	0.01	ns

Data shown are at the plot-scale (4 m^2^) pooled across tree or shrub species within each planting type. Plot ID and sampling periods were included as random effects in models.

*Ns* not significant.

### Invertebrate functional groups differ between shrubs and trees and among vegetation structure treatments

**Figure 3:**
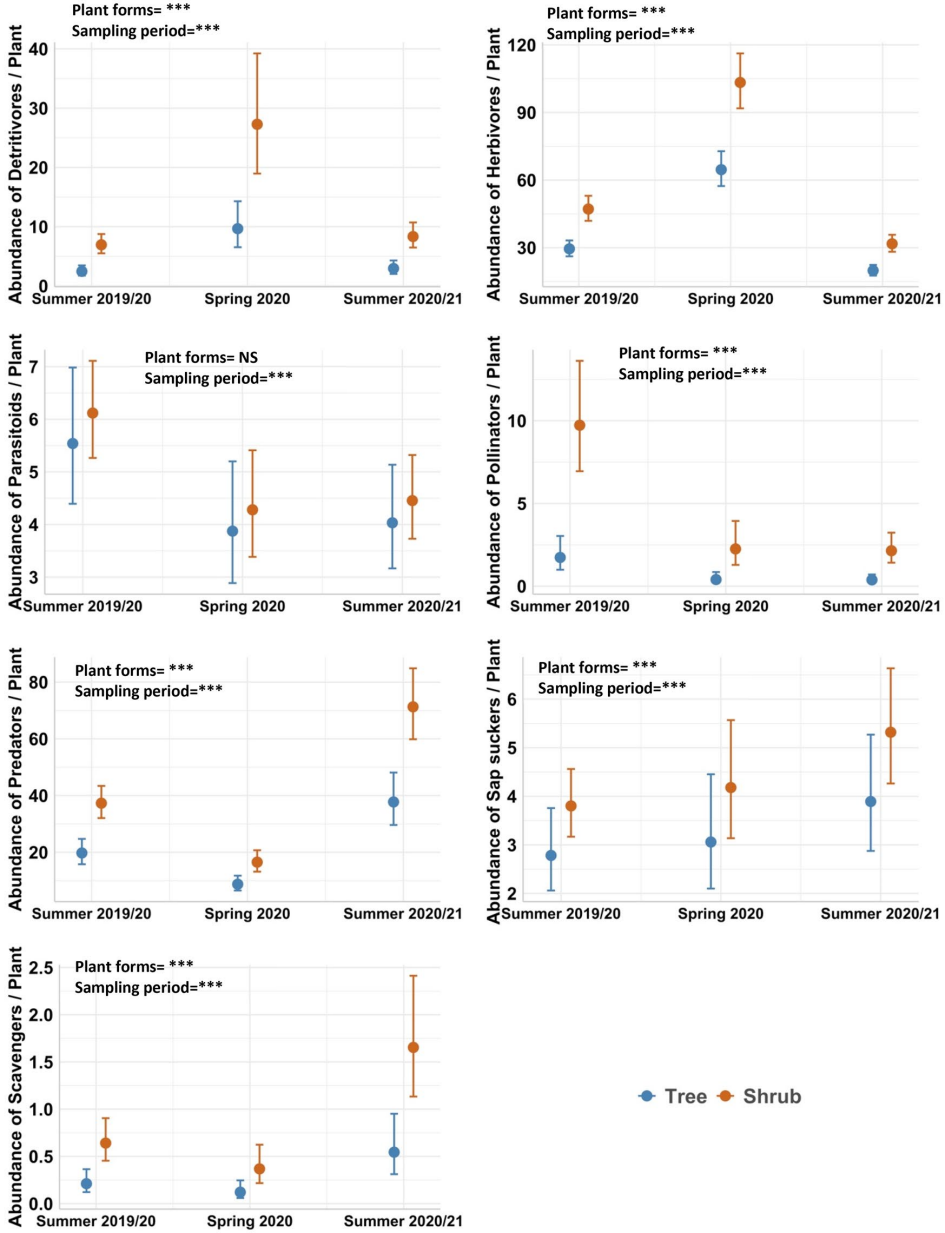
Pairwise comparisons of the abundance of seven invertebrate functional groups between individual plants of shrubs and trees pooled across the “tree only”, “shrub only” and “tree and shrub” vegetation treatments. Circles and error bars depict means and standard errors, respectively (*n* = 32 for trees; *n* = 128 for shrubs). See supplementary Table S5 for ANOVA results of the model for each functional group. Plot ID was included as a random effect. Asterisks represent statistical significance: ***P < 0.001.

**Figure 4:**
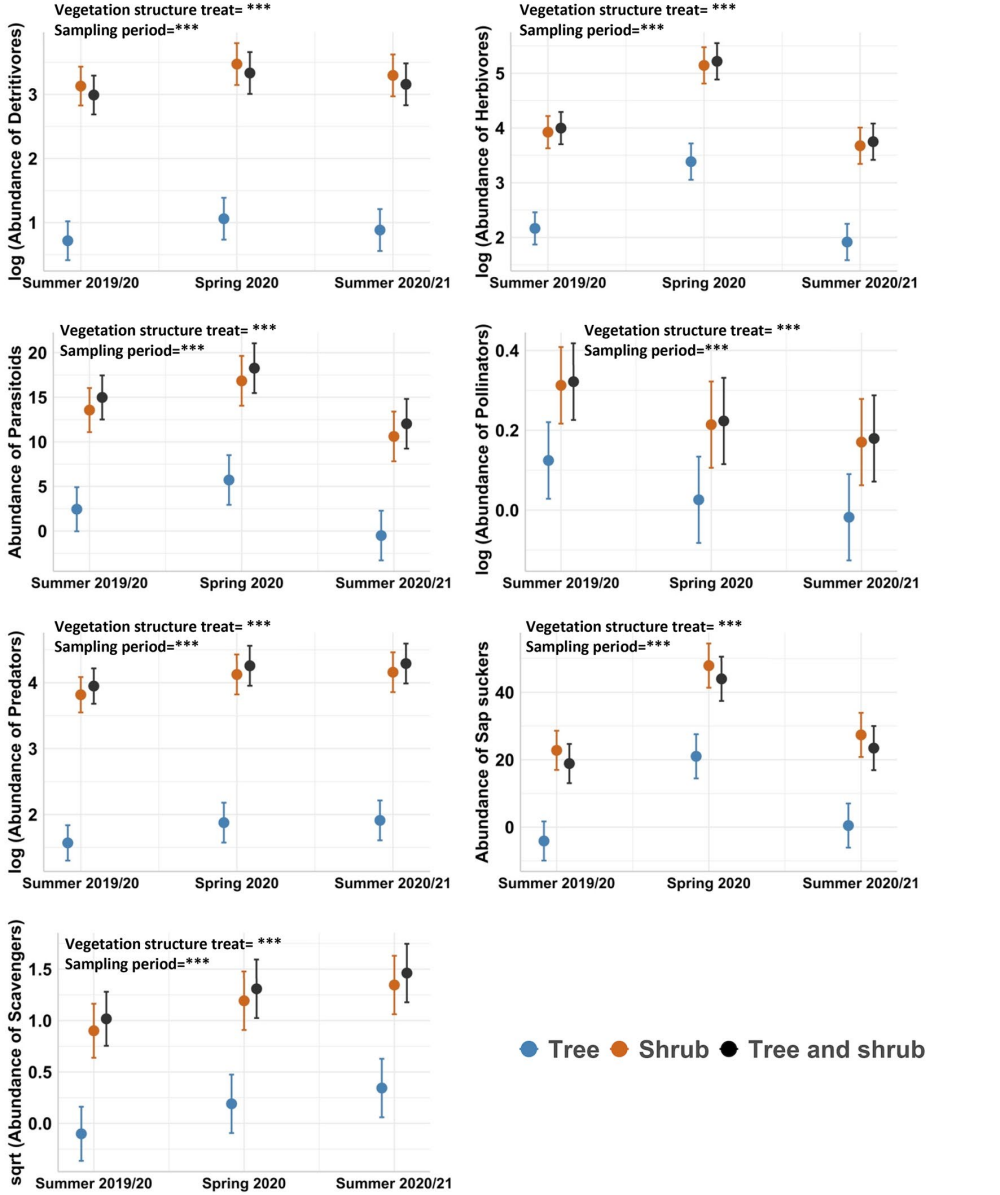
Comparison of the abundance of seven invertebrate functional groups among plot-level vegetation type treatments, across sampling periods—summer 2019/20, spring 2020 and summer 2020/21. Data shown are at the plot-scale (4 m^2^). Circles and error bars depict means and standard errors, respectively (*n* = 16 plots). See supplementary Table S6 for ANOVA results of the *lmer* model for each functional group. Plot ID was included as a random effect in models. Asterisks represent statistical significance: ***P < 0.001.

We used *lmer* models, with plot ID as a random effect, to evaluate variation in the abundance of invertebrate functional groups among individual plants of shrubs or trees and, separately, across different vegetation type treatments. Herbivores and predators were the most abundant groups associated with individual shrubs and trees, and at the plot-level in all vegetation structure treatments, while scavengers were the scarcest group across the full dataset (Supplementary Table S4). Overall, there was a greater number of distinct functional groups (i.e., higher functional group richness) associated with shrubs compared to trees, and in ‘shrub only’ and ‘tree and shrub’ vegetation treatments compared to tree only plots, except for parasitoids which had similar numbers for trees and shrubs (Figs. 3 and 4; Supplementary Table S4, S5 and S6). Detritivores, herbivores, parasitoids and sap suckers were more abundant in spring compared to both summer sampling periods for individual shrubs and trees. The abundance of predators and scavengers was greater in summer 2020/2021 than in the preceding sampling periods for both individual shrubs and trees and for the different vegetation type treatments. In contrast, pollinators were most abundant in summer 2019/20 across these plant types (Figs. 3 and 4; Supplementary Tables S5 and S6).

### Invertebrate functional groups in relation to vegetation traits

For both shrubs and trees, the number of detritivores, pollinators, herbivores and parasitoids on individual plants increased with increasing canopy volume and the presence of flowers. Plant canopy volume explained the majority of data variance for herbivores and parasitoids. The numbers of predators, sap suckers and scavengers showed significant correlations with all measured plant traits (Supplementary Table S7).

For vegetation type treatments, the number of flowering plants in a given plot was the most influential factor explaining the differences in all invertebrate functional groups, except pollinators and parasitoids. Moreover, total LA and total canopy volume were the important explanatory variables for the number of pollinators and parasitoids, respectively (Supplementary Table S8).

## Discussion

Our common garden comparison of tree and shrub planting combinations revealed the significance of shrubs for promoting invertebrate diversity. We showed that shrubs foster high levels of invertebrate abundance as well as greater taxonomic and functional group richness, whether they are planted alone or in combination with trees. However, inclusion of shrubs with trees did not result in a further increase in the number of invertebrates, relative to shrubs growing on their own. Furthermore, we also showed that shrub plantings with wide and dense canopies harboured a greater taxonomic and functional group diversity than those with smaller or more open canopies.

Our study demonstrated that herbivorous taxa, such as moths, trichopterans and thrips were strongly associated with shrubs in a humid sub-tropical peri-urban environment. This finding is in line with a Californian desert study conducted by Zuliani, et al.^34^ which showed that shrubs host a greater abundance and diversity of vertebrate and invertebrate species, compared to trees. While shrubs are generally smaller in height and canopy volume, they typically have multiple stems and comparatively more branches than young trees, reflecting a contrasting crown architecture^35,36^ and more rapid formation of microenvironments underneath their canopies near the ground surface in the early years following establishment. Such characteristics enable shrubs to offer protection from extreme weather conditions by providing a buffer against inclement temperatures and wind^34^. This, together with their greater physical proximity to the ground layer that facilitates movement between soil-, litter- and canopy-dwelling invertebrate communities^34^ may explain the relatively greater abundance and diversity of invertebrate communities associated with shrubs, compared to trees.

The importance of mid-story vegetation, notably shrubs, for sustaining high invertebrate biodiversity—a highlight of our study—has previously been reported by Mata, et al.^5^ for urban parks in Melbourne, Australia. Furthermore, in their field experiment with artificial plants, Grof-Tisza, et al.^37^ showed that dragonfly and damselfly abundance and taxonomic richness were more strongly correlated with the density of branches than vegetation height and width. Herbivores living on larger trees can disperse from one tree to another when individual tree crowns are adjacent to each other. While our study focussed on young trees whose crowns were not fully formed and may not, therefore, reflect mature street trees or urban forest, our findings are of particular relevance for newly established urban areas or new re-vegetation projects, while vegetation is establishing. Hence it is plausible to speculate that invertebrate abundance and richness are likely to increase, and relationships with tree traits become more pronounced, as trees get older. However, it is worth noting that street trees die quite young compared to trees in natural forests. Indeed, it has been estimated that only half of planted street trees will survive for over 20 years^38^.

Despite strong differences in invertebrate communities associated with trees and shrubs, the current study provided little evidence of an association between vegetation structural complexity and invertebrate diversity. The incorporation of shrubs alongside trees did not significantly enhance abundances or richness on invertebrates, relative to shrub-only plantings, which is contradictory to the results of Peng et al.^32^, Tews et al.^39^. The study of Lassau, et al.^17^ in Sydney sandstone ridgetop woodland, showed that pitfall-trapped beetles (but not those caught in-flight) increased with vegetation complexity in habitat comprising both trees and shrubs. Therefore, it is plausible that inclusion of alternative sampling methods (e.g. pitfall trapping or sweep-netting) could offer a more nuanced understanding of the role of vegetation type and/or structural complexity in invertebrate community structure.

The greatest invertebrate abundance and richness of taxonomic and functional groups in both trees and shrubs occurred during flowering periods. In the ‘shrub only’ and ‘tree and shrub’ treatments, the four shrub species had flowers in at least three (out of seven) of the sampling periods and showed a succession in flower emergence (overlapping flowering phenology between species). Wardhaugh et al.^40^ explored the interaction between flowers and invertebrates in an Australian tropical forest and observed that the microhabitat of flowers attracted a greater proportion of invertebrates than tree crowns without flowers. Diverse land uses within cities can promote richness in flowering plant species^41^ and can attract a greater abundance of pollinators than areas with few floral resources, such as agricultural land^42,43^. Thus, diversification of residential land use as well as selection of appropriate flowering plants for urban street plantings has the potential to increase the availability of resources for urban fauna, thereby supporting a broad range of ecosystem services in urban environments.

### Implication for urban greenspace management

This study highlights the need for more refined urban planting strategies that incorporate not just aesthetics and cooling benefits as selection criteria for plants, but also food, shelter, oviposition and nesting site requirements of target invertebrate groups. We showed that shrubs can support high levels of invertebrate biodiversity, as well as a greater abundance and diversity within key functional groups, such as detritivores, predators and herbivores. High invertebrate abundance, in turn, attracts and provisions associated fauna, such as birds and small mammals, thereby potentially enhancing the overall abundance and diversity of other taxonomic groups.

Shrub plantings may be feasible in parks, roundabouts and urban gardens, although in more heavily built environments, concrete walls or pavements and streetscape visibility considerations may constrain planting options. However, where shrubs can be planted, the additional floral and trait diversity and associated microhabitats are likely to attract a greater abundance and diversity of invertebrates. Maintaining shrubs in urban areas does, however, have challenges, including water scarcity, competition with trees for water and soil resources, air pollution and vandalism. These challenges may be overcome by revising urban greening strategies—which typically focus on trees^44–46^—to facilitate the establishment, maintenance and sustainability of shrubs (e.g., through establishment of planting beds with improved soil properties and design). By broadening planting strategies and policy to incorporate shrubs in addition to trees, urban environments can benefit not just from an aesthetic perspective, but also in terms of their biodiversity and ability to provide sustainable ecosystem services.

### Study constraints and considerations

Use of both native and non-native trees and shrubs in urban plantings is commonplace worldwide^47,48^. Given the established relationship between invertebrate biodiversity and native plant species^49,50^, the inclusion of two native tree species among the four tree species, in comparison to four native shrubs, has the potential to influence the tree *vs*. shrub comparison. Similarly, recognizing the high degree of specificity in arthropods towards the host taxa they consume, the inclusion of three members from the same family among the native shrubs could influence the observed patterns and make it more challenging to extrapolate findings. However, statistical comparison between native and exotic trees in our study (Supplementary Fig. S2) indicated that invertebrate abundance and richness did not differ between these groups when trees were in leaf, and that differences were only evident when exotics (which are deciduous) had no leaves (in early spring). This indicates that the origin of the selected tree species likely had little impact on observed differences in invertebrate abundance and richness between shrub and tree plots. A similar lack of differences in invertebrate communities associated with native and exotic vegetation has also been reported for forests^52^ and urban gardens^51^.

While vegetation structure is an important feature of both shrubs and tree species’ innate growth characteristics, management interventions such as pruning may also be important. As plants grow in height and canopy structure, relationships with invertebrates may change over time. Studies have consistently demonstrated the critical role that trees, especially mature trees, play in providing a wide range of microhabitats (such as tree hollows, snags, more textured bark) for invertebrates^35^. The relatively young age (3 years) of the trees in our study may have contributed to the lower abundance and taxonomic richness of invertebrate communities on these, relative to same-age shrubs. However, Gotmark, et al.^36^ clearly note the advantages of a small shrub compared to a small tree with equivalent above-ground woody volume, with the former having a larger cross-sectional stem area, a greater area of photosynthetic tissue in bark and stem and a greater area for sprouting. These are all features that can provide varied microhabitat for associated species. Nonetheless, our findings reflect patterns expected in the early years as tree and shrub plantings are establishing in urban areas. Acknowledging these nuances is crucial for refining future studies and enhancing our comprehension of vegetation-invertebrate ecological dynamics in the context of urban biodiversity and ecosystem functioning.

## Materials and methods

### Study site

In order to understand how the complexity of urban vegetation can influence insect abundance and diversity, we established a common garden experiment comprising young trees and shrubs, growing on their own or together, in the peri-urban setting of the Hawkesbury campus of Western Sydney University, in Richmond, New South Wales (NSW), Australia (37° 49′ 22.8072'' S, 144° 59′ 52.8036'' E). The study was conducted during the austral summer and spring across two consecutive years (summer 2019/20, spring 2020 and summer 2020/21). The selected sampling timeframes were based on periods with significant invertebrate activity. Weather conditions at the study site during survey periods are summarized in Table 3.

**Table 3:** Climate at the study site and weather conditions during invertebrate survey periods (2019–2021).

	1960–90 Average^52^	Summer 2019/20^53^	Spring 2020^54^	Summer 2020/21^55^
Mean maximum temperature (°C)	23.7	31.4	25.2	27.9
Mean minimum temperature (°C)	17.5	18.2	15.9	17.0
Total rainfall (mm)	906.3	320	174.6	268

### Data collection

#### Study design

**Figure 5:**
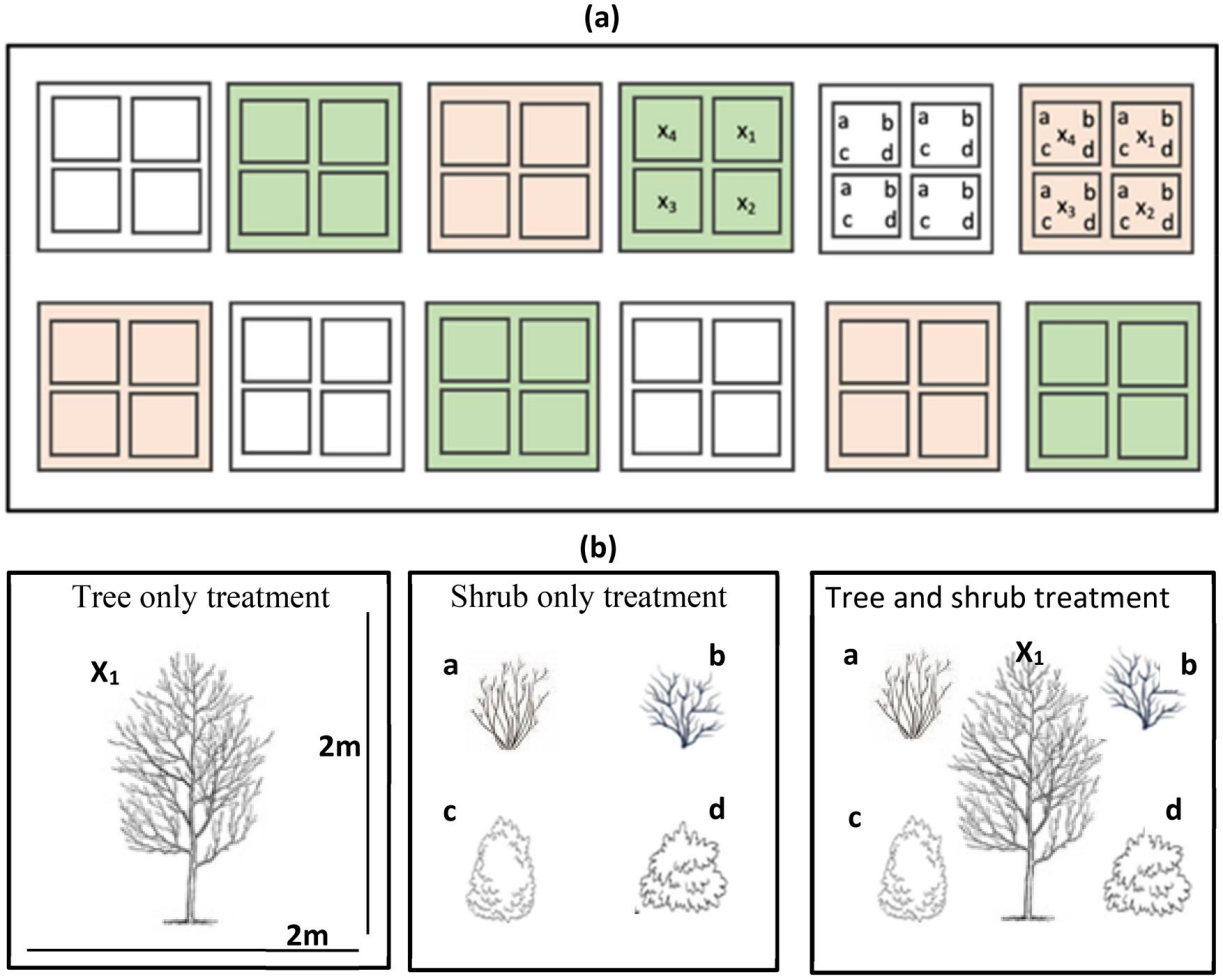
Study design of this research. The site comprises (**a**) three vegetation type treatments—‘tree only’ (green colour), ‘shrub only’ (white colour) and ‘tree and shrub’ (orange colour), each replicated four times in a randomised design. Each vegetation type treatment replicate has nested within it four plots of 4 m^2^ area each. (**b**) Only one 2 × 2 m plot for each treatment type is displayed in the drawings. The ‘tree only’ treatment has one of the four tree species (x_1_, x_2_, x_3_, x_4_) planted in each 4 m^2^ plot, ‘shrub only’ has all four shrub species (**a**–**d**) in each 4 m^2^ plot and ‘tree and shrub’ has one tree species and all four shrub species in each 4 m^2^ plot. For ‘tree only’ and ‘tree and shrub’ only one tree species (x_1_) is displayed in the drawings. Species names and phenology are given in Table 4.

Four tree species (eight individuals per species) and four shrub species (32 individuals per species) were used for the experiment (Fig. 5; Table 4). All species are commonly used in urban plantings across Australian cities; two trees—*Lophostemon confertus* and *Elaeocarpus reticulatus—*are native to north-east NSW; two tree species—*Lagerstroemia indica* and *Liriodendron tulipifera* are exotic to Australia. All four shrub species—*Baeckea virgata*, *Melaleuca citrina*, *Melaleuca thymifolia* and *Westringia fruticosa* are native to NSW^56,57^. The dominance of the Myrtaceae family (one tree species and three shrub species) in the experimental setup reflects its dominance in urban landscapes in Sydney^58^. The experimental site covered an area of ~ 3900 m^2^ and was composed of 48 plots, each 2 × 2 m. Plots were arranged in four replicate blocks, each comprising 12 plots. All the plots were growing in isolation and were located at a distance of 4 m from each other and at least 30 m distance from other woody vegetation. The spatial context of the experimental design is illustrated in Supplementary Fig. S3. The experiment complied with relevant institutional, national and international guidelines and legislation.

**Table 4:** List of tree and shrub species and families used in this study and origin of each species (N = native to Australia, E = exotic).

	Family (origin)	DEC 2019	JAN 2020	FEB 2020	SEP 2020	NOV 2020	DEC 2020	JAN 2021	Height (cm) (mean + se)
		Summer 2019/2020			Spring 2020		Summer 2020/2021		
Tree species									
*Elaeocarpus reticulatus*	Elaeocarpaceae (N)	x				x	x		347 (25.4)
*Lagerstroemia indica*	Lythraceae (E)	x	x	x		x	x	x	478 (21.9)
*Liriodendron tulipifera*	Magnoliaceae (E)								309 (51.5)
*Lophostemon confertus*	Myrtaceae (N)	x				x			253 (8.5)
Shrub species									
*Baeckea virgata*	Myrtaceae (N)	x	x				x	x	183 (2.6)
*Melaleuca citrina*	Myrtaceae (N)				x	x	x		148 (2.7)
*Melaleuca thymifolia*	Myrtaceae (N)					x	x	x	63.7 (2.9)
*Westringia fruticosa*	Lamiaceae (N)				x		x	x	98.9 (4.7)

The presence of flowers for each species during the survey times is marked with an “x”. Means and standard errors of the plant height (cm) in January 2021 are given for individuals pooled across all planting combinations.

Trees (in 45 L bags) and shrubs (in 140 ml pots) were sourced from a local plant nursery; all tree stock conformed to the Australian Standard AS-2303^59^. Trees and shrubs were planted at the end of October 2018. Individual trees were planted into 60 cm deep and wide holes with the addition of a slow-release fertiliser mixed into the soil around the root ball (NPK 21.8:0.7:7.2, Osmocote Fertiliser; Scotts Australia). Each plot had an area of 4 m^2^ and was in a grass matrix, resembling nature strip on park settings. Woodchip mulch, at a depth of 100 mm, was applied across the 4 m^2^ plot. Although all tree individuals were of the same age at the time of planting, differences in tree height were recorded (average heights: *L. confertus* 1.87 m ± 0.15; *E. reticulatus*, 1.79 m ± 0.49; *L. indica* 1.62 m ± 0.94 and *L. tulipifera* 1.03 m ± 0.11; *n* = 8 individuals per species). Shrub species also showed differences in height at the time of planting (average heights: *B. virgata* 39.53 cm ± 8.39; *M. citrina* 41.06 ± 8.06; *M. thymifolia* 26.84 ± 5.67 *and W. fruticosa* 13.75 ± 3.75; *n* = 32 individuals per species). All plots were watered individually using 2 × 8 L h^−1^ drippers. During the first four weeks of establishment, plants were irrigated every two days. Beyond this period, plants were irrigated every seven days until October 2019, when this was increased to every four days until the end of April 2020 due to the dry conditions in the study area during summer 2019–2020. Irrigation then returned to a weekly schedule at the end of summer 2020. On irrigation days, water was supplied over a 40-min period (ca. 10.7 L day^−1^).

The experiment comprised three levels of a vegetation type treatment namely ‘tree only’ (1 individual of 1 species), ‘shrub only ‘(4 individuals, 1 each of 4 species) and ‘trees and shrubs’ (5 individuals—1 tree species, 4 shrub species) plantings. The ‘tree only’ treatment was replicated four times for each individual species (i.e. a total of 16 plots). The ‘shrub only’ treatment was composed of four individuals, one of each of the four species, replicated four times in each block, over four blocks (giving a total of 16 plots). The ‘tree and shrub’ treatment was composed of four individual shrubs—one of each species—and one individual tree, repeated for each of the four tree species. Each of these was replicated four times, giving a total of 16 plots (Fig. 5). Throughout the experimental period, individual plant crowns remained distinct from each other.

#### Invertebrate sampling

Invertebrate samples were collected from individual plants using the branch-beating method, which mainly targets less-mobile taxa. The sampling regime was determined based on observations from a pilot study undertake prior to the main study fieldwork. For this, the whole of each individual plant’s canopy was beaten for 60-s, 40-s and 20-s, and the observed abundance and richness of invertebrates were compared. This assessment revealed that the 20-s beating captured over 80 percent of the invertebrates captured in the 60-s samplings.

**Figure 6:**
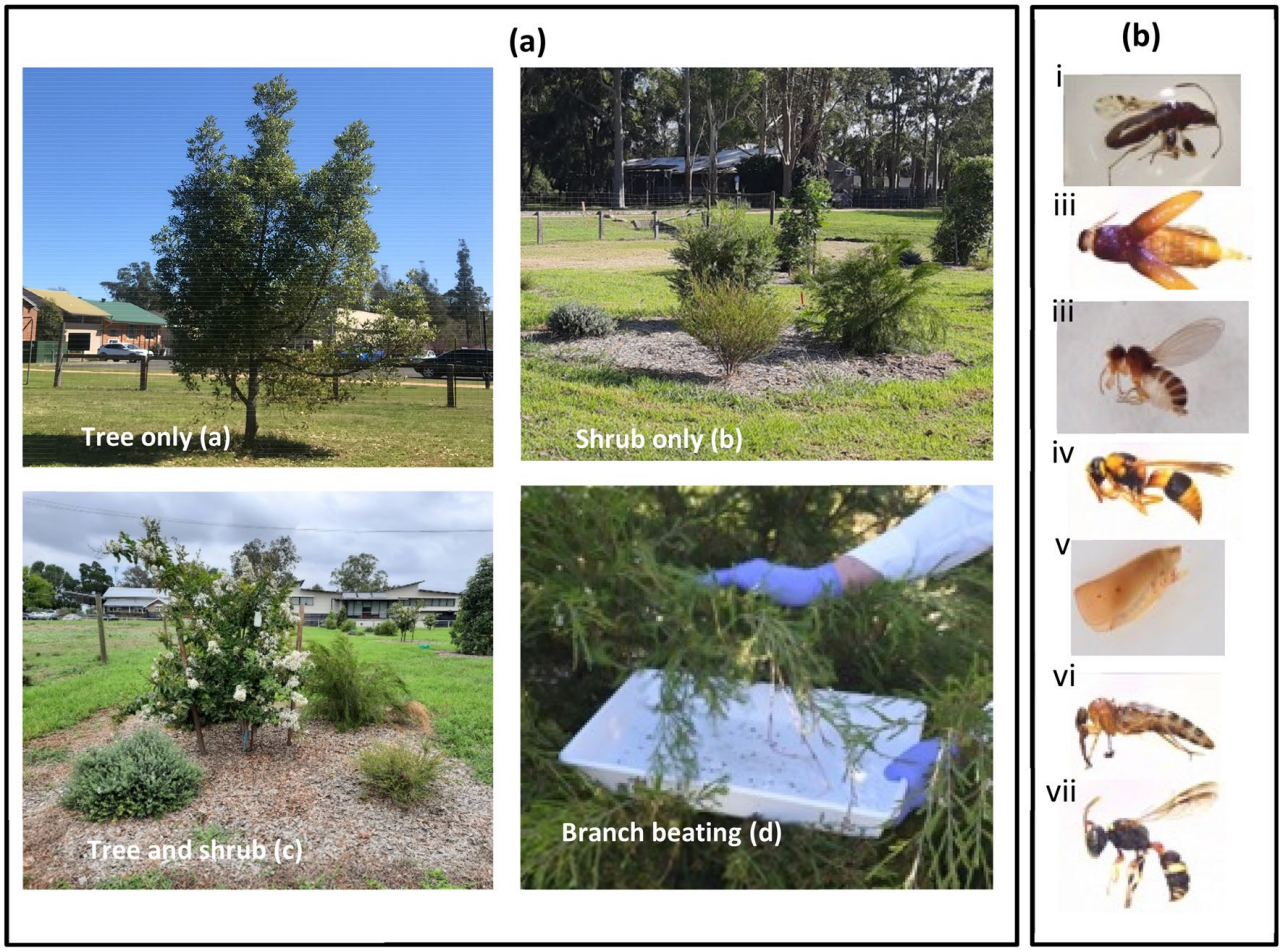
Panel (**a**) shows the experimental design, with examples of ‘shrub only’, ‘tree only’ and ‘tree and shrub’ treatment plots (**a**–**c**) and invertebrate sample collection (**d**). Panel (**b**) shows example photographs taken during the early summer 2020/21, following two years of site establishment. Examples of insects collected: Rhyparochromidae (Hemiptera); (ii) Elateridae (Coleoptera), (iii) Chloropidae (Diptera), (iv) Vespidae (Hymenoptera), (v) Flatidae (Hemiptera), (vi) Asillidae (Diptera), (vii) Crabronidae (Hymenoptera).

Samples were collected on two white trays (45 × 30 cm). Trays were placed under the plants and branches were shaken for 10 s, by two people on opposite sides of the plant (i.e. a total of 20 s per plant). Trays had 150 ml of water containing a drop of detergent in them to prevent invertebrates from escaping. Individuals collected from both trays were combined and transferred to 70% ethanol-filled containers for storage and subsequent identification. Sampling was conducted only under optimal weather conditions (i.e., days with clear sky, 1–1.4 m s^−1^ wind speed and warm temperatures, ≥ 24 °C) when insects were more active. Invertebrate sample collection was carried out on 2–3 consecutive days, from 09:00 to 15:00, for each sampling period (Table 3). To minimise the effect of surrounding vegetation, spaces between the sampling plots were mown one week before every sampling period (Fig. 6).

#### Invertebrate identification

Invertebrate identification was performed in two stages. First, ants, spiders and springtails were separated from the samples, identified to morphospecies and counted. Following this, the remaining samples were identified to family level using identification keys^60^. Immature stages were also considered in the identification process and those that were not identifiable were grouped as larvae/nymphs/pupae. Subsequently, invertebrates were categorized into seven functional groups: detritivores, herbivores, parasitoids, pollinators, predators, sap suckers and scavengers (Supplementary Table S1). All specimens were stored in the Entomology Lab of the Hawkesbury Institute for the Environment, Western Sydney University, Richmond, NSW, Australia.

Plant traits i.e., plant height (cm), canopy width (m), canopy depth (m) and leaf area index (LAI) (m^2^m^−2^) were measured during each sampling season. Tree height and crown depth were measured using a Haglöf laser meter (L400, Haglöf, Sweden) with a height resolution of 0.01 m. Canopy width measurements were taken as the length of *x* and *y* orthogonal axes from edge to edge through the crown centre and then averaged. LAI was measured for each individual plant using a plant canopy analyser (LAI-2200, Li-COR, USA). LAI is the ratio of the area of leaves to the area of the ground under the crown and was measured under diffuse light on overcast days. Four LAI measurements were performed from four corners of each plant at 1 m height above ground for trees and 10 cm height for shrubs, and with a 90° view cap on a fish-eye lens. LAI data were analysed using FV2200 software developed for LAI-2200, deploying an isolated crown model and removing the 5th mask (68°) to remove the effects of any surrounding objects; these four measurements were averaged for each plant. The presence or absence of flowers for each plant was documented during sampling periods. To calculate plant canopy volume (*v*), all individual plants were considered as a cylinder^61^. Equation (1) was used to calculate plant canopy volume, where *h* and *r* denote height and canopy width, respectively. Leaf area per plant (LA) was calculated using Eq. (2), where LAI and PCA denote leaf area index and projected crown area (PCA), respectively. The projected crown area (PCA; m^2^) was calculated as an ellipse using crown diameters in the wider and narrower directions^62^. Canopy volume and LA across all plants within a plot were summed to obtain plot-level values.

1 $$V = \pi r^{{\mathbf{2}}} h$$

2 $$LA \, = \, LAI \, / \, PCA$$

### Data analysis

Invertebrate count data were used to calculate the species-level abundance and taxonomic richness of invertebrate communities and their associated functional groups. Data analyses were conducted first to evaluate differences between trees and shrubs and then to evaluate differences among the different vegetation type treatments (i.e., ‘shrub only’, ‘tree only’, ‘tree and shrubs’). Before pooling data for individual plants by tree and shrub groups across the various planting combinations, we tested the differences in invertebrate abundance and richness between trees and shrubs when they were growing alone and when growing in combination (i.e., tree only *versus* tree and shrub; shrub only *versus* tree and shrub). These tests revealed no significant differences in planting combinations in invertebrate abundance or richness for shrubs (abundance, t = 0.89, *p* = 0.37; richness, t = 0.40, *p* = 0.69) or trees (abundance, t = 0.21, *p* = 0.84; richness, t = 0.68, *p* = 0.50) (Supplementary Fig. S1). Invertebrate abundance associated with native trees was compared with exotic trees revealing differences between native and exotic species (abundance, *p* = 0.00; richness, *p* = 0.00) were significant in spring 2020, when exotic trees were leafless (Supplementary Fig. S2). Differences between native and exotic species were not significant for summer 2019/2020 (abundance, *p* = 0.49; richness, *p* = 0.66) or summer 2020/2021 (abundance, *p* = 0.89; richness, *p* = 0.98). Invertebrate abundance and richness among individual tree and shrub species are compared in Supplementary Fig. S4.

To assess sampling effort and compare taxonomic richness for both plant types and vegetation structures across the three sampling periods, invertebrate species accumulation curves were plotted (Supplementary Fig. S5). The function *specaccum*, which is a sample-based rarefaction method from the Vegan package^63^, was used to produce species accumulation curves for each season. To compare the abundance and richness of invertebrates (as response variables) between trees and shrubs, data were analysed using Fit Generalized Linear Mixed Models with the *glmerPQL* function from the MASS package^64^. Plant type (i.e., tree or shrub) and sampling periods were used as explanatory variables and species ID nested with plot ID were considered as random effects in this model (Table 5).

**Table 5:** Models used to analyse invertebrate data.

	Function	Response variable	Explanatory variable	Random effect
Question 1: To investigate whether shrubs will support a greater abundance and taxonomic richness of invertebrates than young trees				
Plant types (trees and shrubs)	*glmerPQL*	Invertebrate abundance	Plant type and sampling round	Species ID nested with Plot ID
	*lmer*	Taxonomic richness		
	*lmer*	Abundance of invertebrates of each functional group		
Plant structure (‘shrub only’, ‘tree only’, ‘tree and shrub’)	*lmer*	Invertebrate abundance	Vegetation structure and sampling round	Plot ID
	*glmer.nb*	Taxonomic richness		
	*lmer*	Abundance of invertebrates of each functional group		
Question 2: To investigate whether shrubs enhance plot-level biodiversity when planted alongside trees				
Plant types (tree and shrub)	*lmer*	Invertebrate abundance	Canopy volume, LA and presence of flower	Plot ID sampling round
	*lmer*	Taxonomic richness		
Plant structure (‘shrub only’, ‘tree only’, and ‘tree and shrub’)	*lmer*	Invertebrate abundance	Canopy volume, LA and number of plants in flower	Plot ID sampling round
	*lmer*	Taxonomic richness		

To evaluate differences in invertebrate abundance (as the response variable) for vegetation type treatments (i.e., ‘shrub only’, ‘tree only’, ‘tree and shrub’), data were analysed using the *lmer* function from the lme4 package^65^; data were natural log-transformed (ln) to ensure normality of errors. To compare invertebrate richness for vegetation structure treatments, the *glmer.nb* function from lme4 package was used^65^. For both models, sampling period (summer 2019/20; spring 2020 and summer 2020/21) was used as a fixed effect and plot ID as a random effect (Table 5).

To evaluate relationships between plant traits and the abundance and richness of invertebrates, both overall and for each functional group, data for individual shrubs or trees and vegetation type treatments (i.e., ‘shrub only’, ‘tree only’, ‘tree and shrub’) were analysed using the *lmer* function in R. Trait variables at the individual plant level (trees and shrubs) including tree height, volume, leaf area per plant (LA) and canopy width are presented in Supplementary Fig. S6. Each model included plot level variables (i.e., treatment, plant volume (sum of the canopy volume of all plants present in each plot), LA and the number of plants in flower) as fixed effects, and sampling round and plot ID as random effects. Models were fitted using the *lmer* and *glmer.nb* functions in the lme4 package^65^. Canopy width and height were not included in the models since both traits were highly correlated (r = 0.91 and 0.81, respectively) with plant canopy volume. Residual plots were inspected to check model fits. For pairwise comparison, post-hoc Tukey tests were conducted using the *Multcomp* package^66^. All analyses were performed using R version 3.2.2^67^ and statistical significance was considered at *p* ≤ 0.05.

### Supplementary Information

Supplementary Information.

## Data Availability

The experimental research of this study did not require collection of plant material of any kind. The datasets used and/or analysed during the current study available from the corresponding author on request.
