# Supplementary material for: Urban greening with shrubs can supercharge invertebrate abundance and diversity
Source: Sci Rep. 2024 Apr 16;14:8735. doi: 10.1038/s41598-024-58909-8 (PMC11021404; doi:10.1038/s41598-024-58909-8)
Supplement: Supplementary file 1 — Supplementary Information. [file 41598_2024_58909_MOESM1_ESM.pdf]

Supplementary Figure

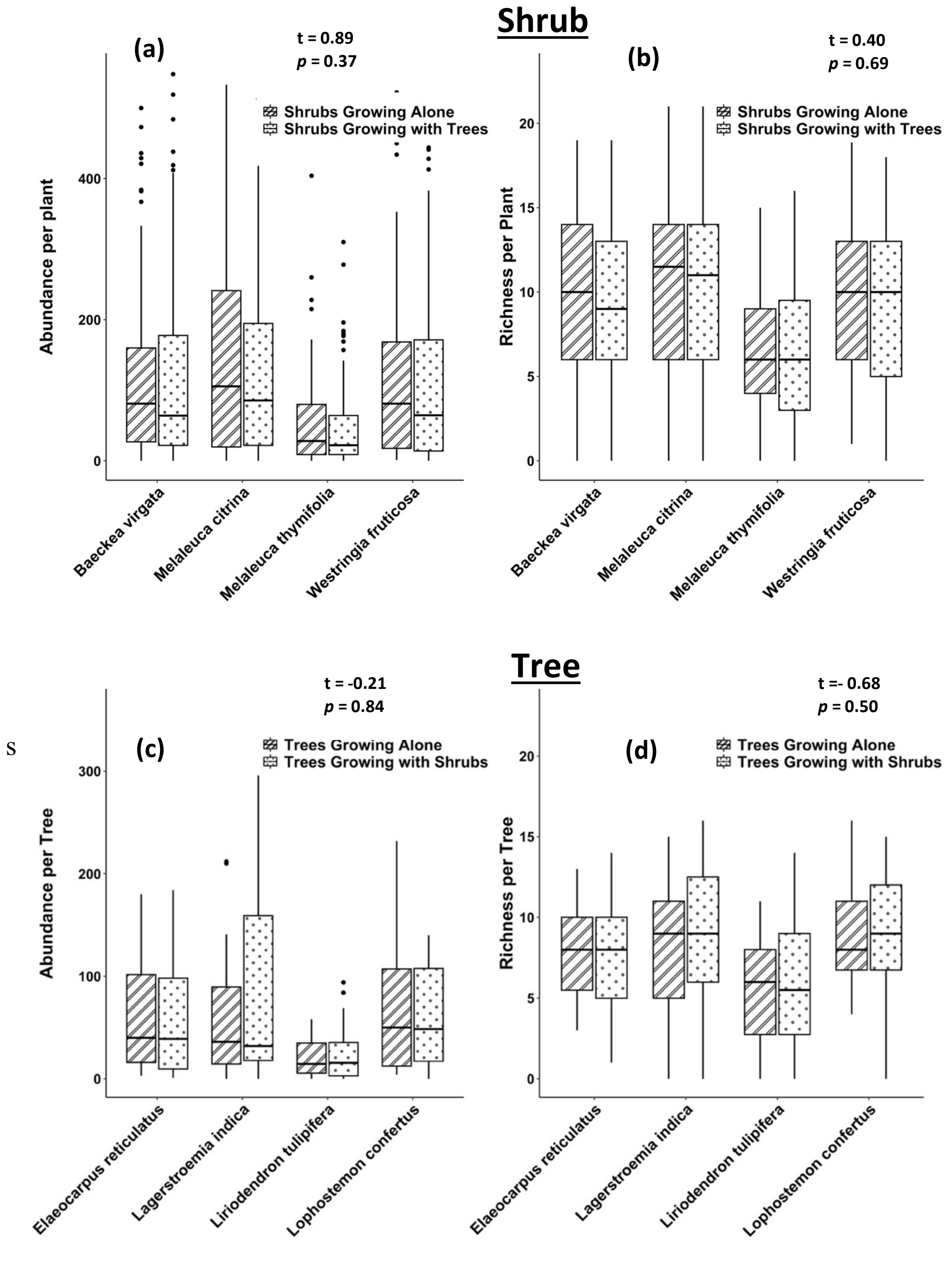

Supplementary Figure 1. Plots show comparisons of invertebrate abundance and taxonomic richness for shrub growing alone (n = 16 plants for each species, n = 64 pooled) and shrub growing with tree (n = 16 plants for each species, n = 64 pooled) respectively (a & b); and tree growing alone (n= 4 plants for each species, n = 16 pooled) and tree growing with shrub (n= 4 plants for each species, n = 16 pooled) respectively (c & d). t-test results of data pooled across tree and shrub species showed that invertebrate abundance and taxonomic richness of tree and shrub did not differ between plants grown alone vs plants grown with each other.

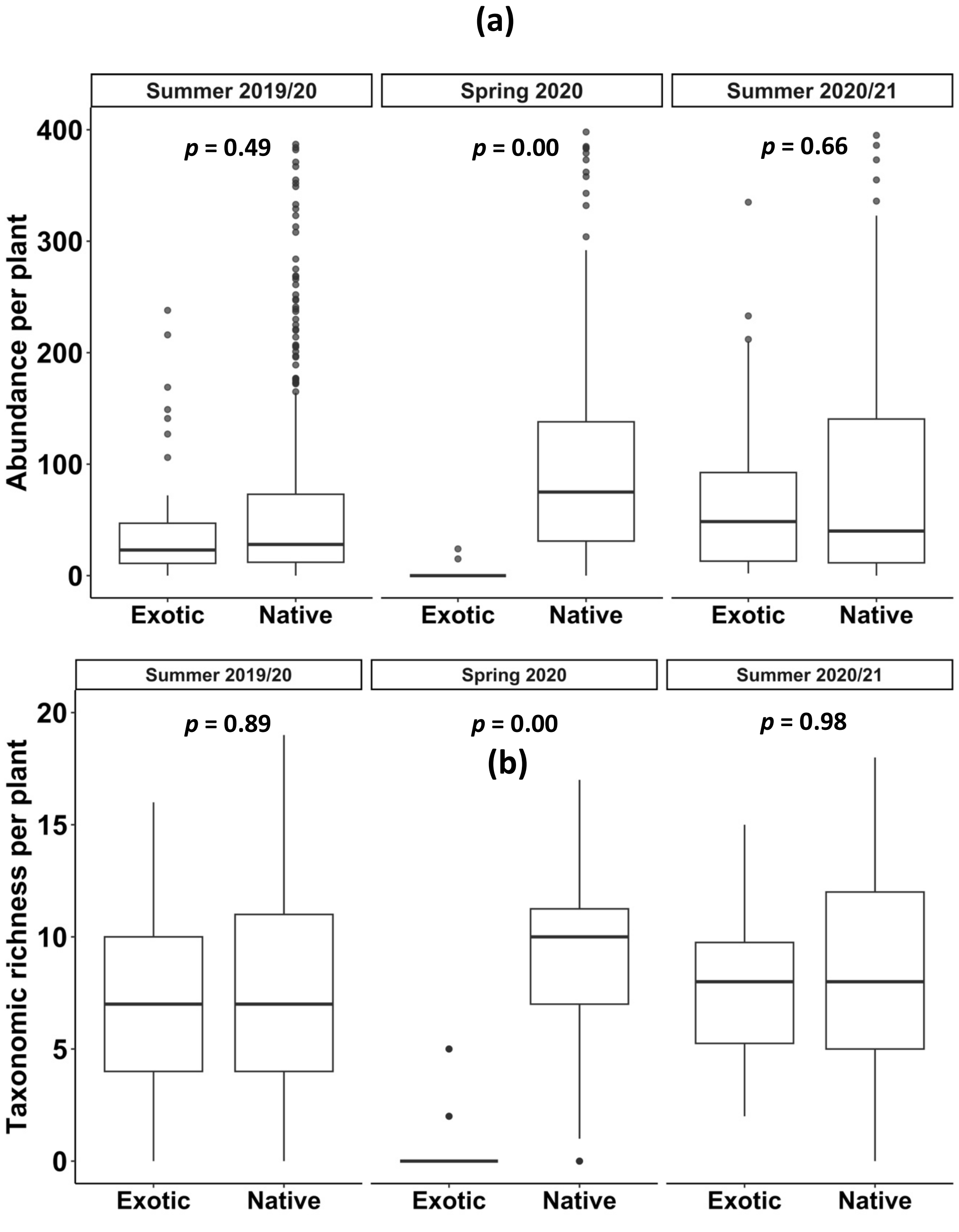

Supplementary Figure 2: Plots show comparisons of invertebrate abundance (a) and taxonomic richness (a) between native and exotic tree species.

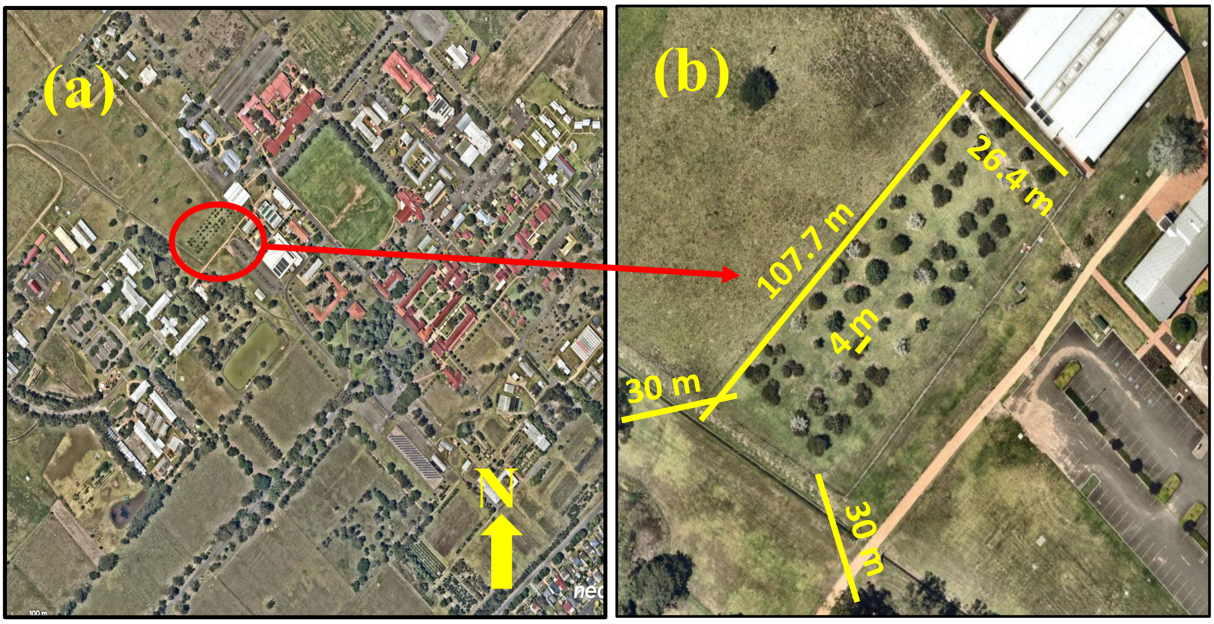

Supplementary Figure 3: Spatial context of the experimental site. The red circle on the left image indicates the location of the experimental site in Richmond (a). The right image indicates the size of the experimental site and the distance of the plots from the surrounding vegetation (b). The dimensions of the site are approximately 107.5 m in length and 26.5 m in width. Each plot measures 4m x 4m and is spaced 4m apart from one another. The surrounding woody vegetation is located at a minimum distance of 30m from the site. (Source: NearMap, Richmond, New South Wales, Australia. http://apps.nearmap.com [January 10, 2024]).

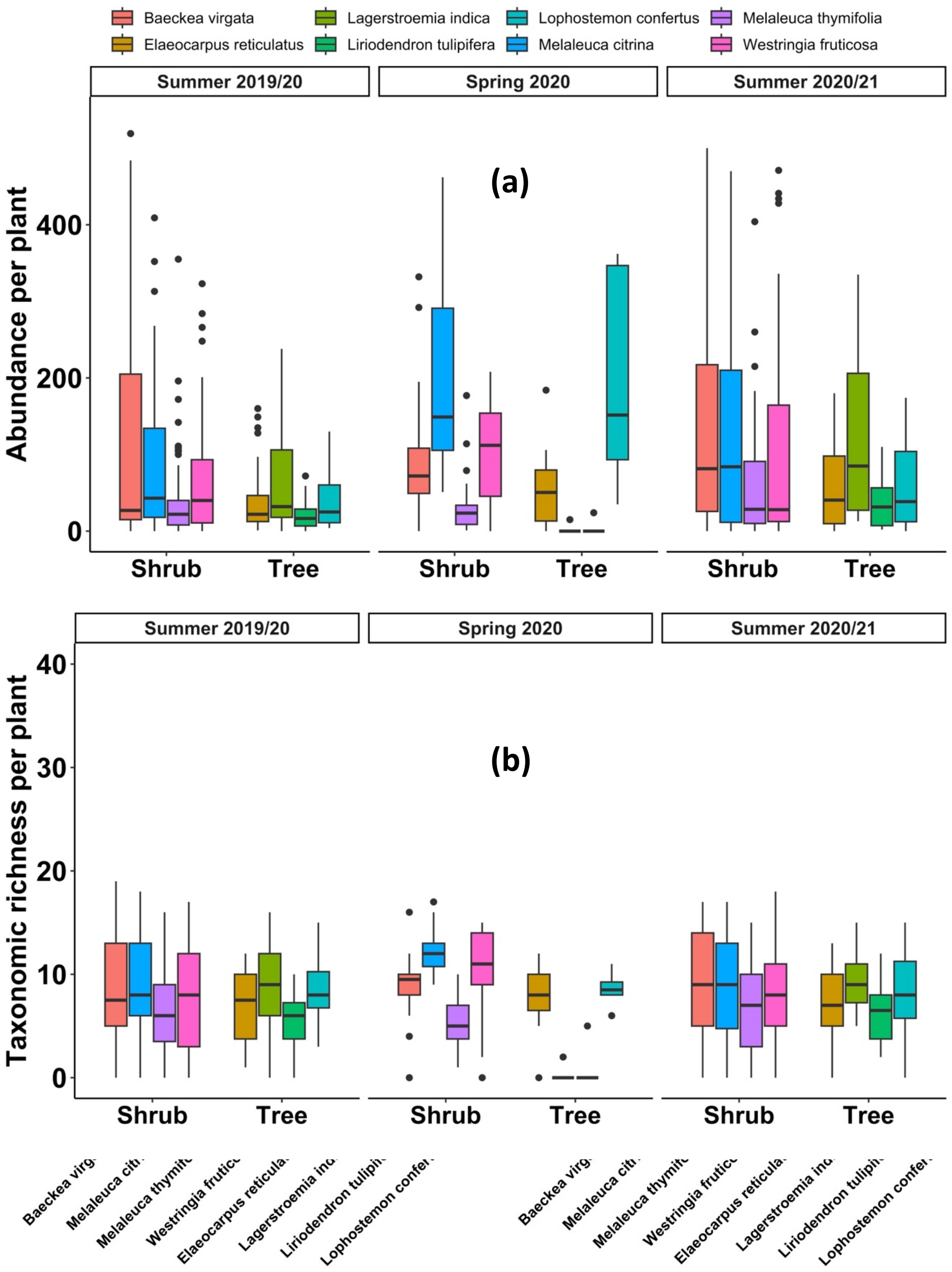

Supplementary Figure 4: Plots show comparisons of invertebrate abundance (a) and richness (b) between tree and shrub species.

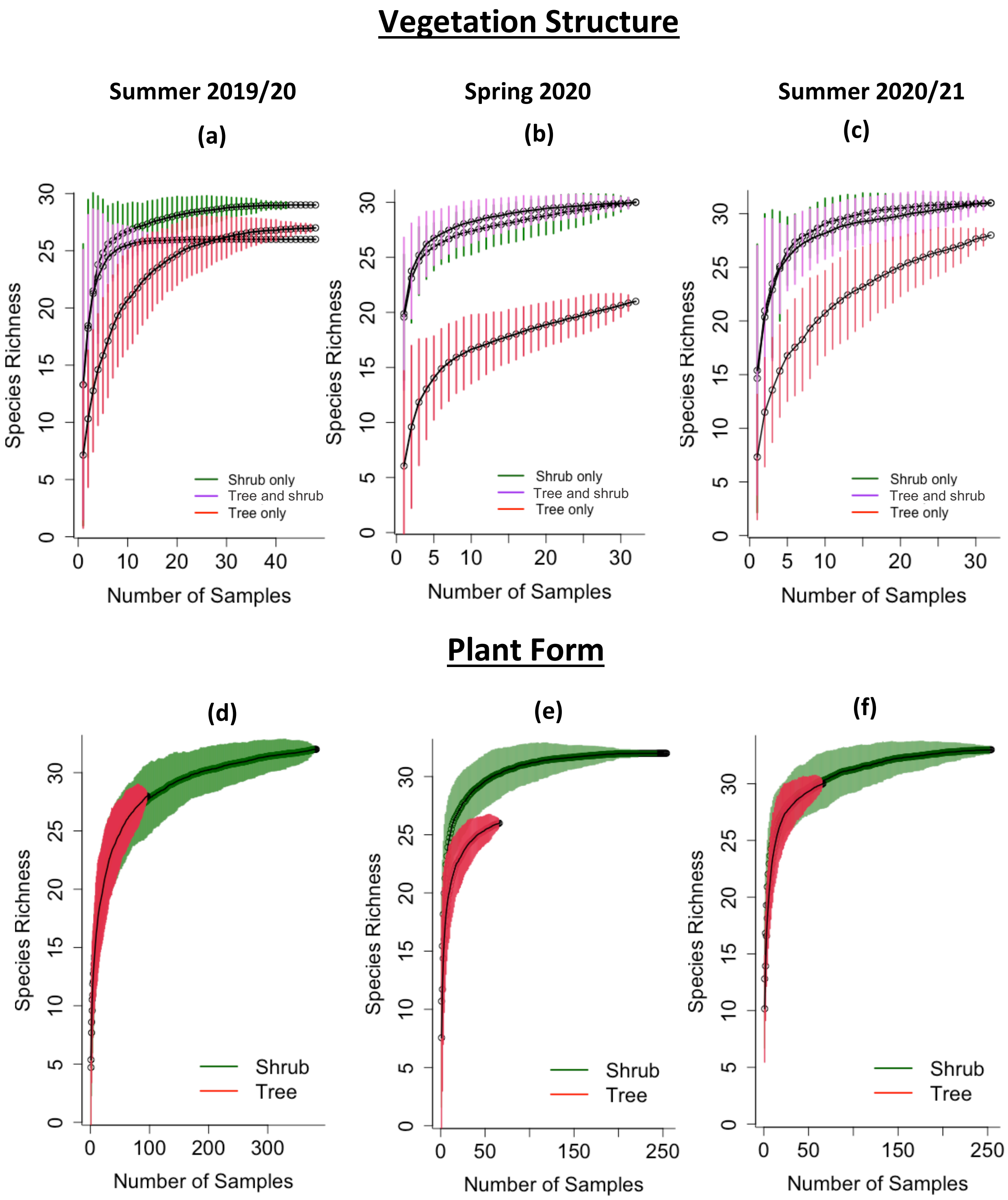

Supplementary Figure 5. Species accumulation curves for all three vegetation structures – ‘tree only’, ‘tree plus shrub’ and ‘shrub only’ (a-c) and plant forms- shrub and tree (d-e) for summer 2019/20, spring 2020 and summer 2020/21.

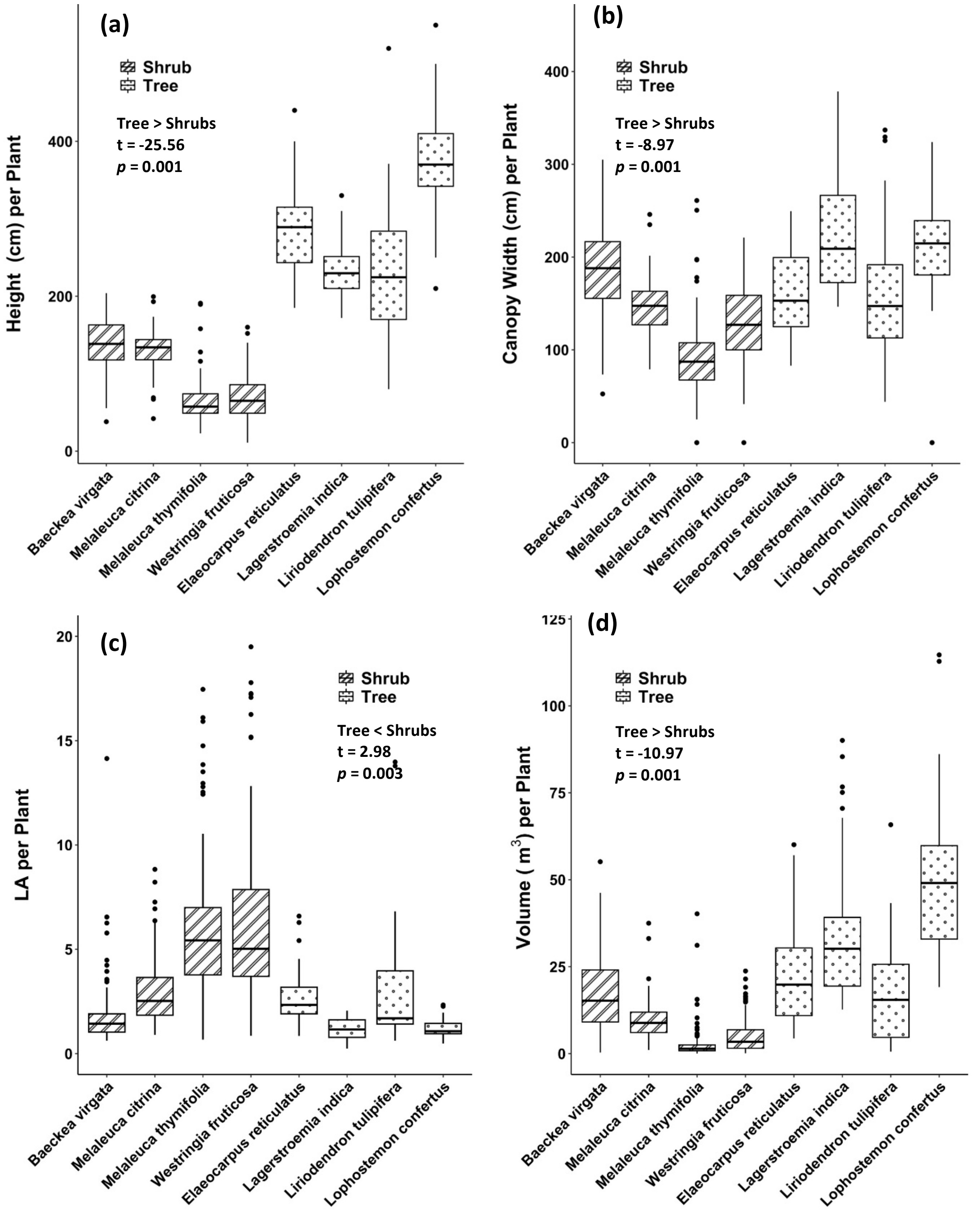

Supplementary Figure 6. Comparisons of height (a), canopy width (b), leaf area per plant (LA) (c) and volume (d) among shrub and tree species (n = 8 plants for each tree species, n = 32 for each shrub species) at the end of the study in January 2021. t-test results of tree and shrub pooled across species showed that plant traits varied significantly between tree and shrub.

Supplementary Table

Supplementary Table 1 Invertebrate functional group categorisations based on Triplehorn, et al. [45].

Functional groups	Definition	Invertebrate groups/families	Order
Detritivores	A group of organisms that feeds on detritus or organic waste.	Formicidae<br>Trichoptera	Hymenoptera<br>Lepidoptera
Herbivores	A group of invertebrates that feeds on plant parts	Nymph/ larva of Moths/<br>Trichoptera<br>Seed wasp<br>Fig wasp<br>Agromyzidae<br>Acrididae<br>Heleomyzidae<br>Argidae<br>Midge<br>Fungus gnat<br>Symphyta<br>Chrysomelidae<br>Chrysomelidae:Alticinae<br>Culicidae<br>Cynipidae<br>Chloropidae<br>Curculionidae<br>Cerambycidae<br>Psocoptera<br>Prostigmata<br>Noctuidae<br>Terebrantia<br>Tubulifera<br>Cricket	Hymenoptera<br>Lepidoptera<br>Coleoptera<br>Heteroptera<br>Orthoptera<br>Thysanoptera<br>Homoptera
Parasitoids	A group of invertebrates that parasitize other arthropods by depositing eggs in the pupae, larva, or eggs of their host.	Bethylidae<br>Brachonidae<br>Chalcididae<br>Sub-Chalcidoidea<br>Ichneumonidea<br>Encyrtidae<br>Eurytomidae<br>Gastropidae<br>Ichneumonidae<br>Megaspilidae<br>Mymaridae<br>Platygastridae<br>Pteromalidae<br>Perilampidae	Hymenoptera<br>Diptera

		Scelioninae<br>Proctotrupidae<br>Torymidae<br>Tachinidae	
Pollinators	A group of invertebrates that transfers pollen from the anther of one flower to the stigma of another, thereby helps plants to reproduce	Honeybees<br>Aculeata<br>Anthicidae<br>Cleridae<br>Colletidae<br>Halictidae<br>Nitidulidae<br>Mordellidae<br>Scarabaeidae<br>Syrphidae	Hymenoptera<br>Coleoptera
Predators	A group of organisms that usually feeds on other invertebrates	Spider<br>Anthocoridae<br>Asilidae<br>Cantharidae<br>Coccinellidae<br>Hemerobidae<br>Mutillidae<br>Melyridae<br>Monotomidae<br>Mesostigmata<br>Mantispidae<br>Nabidae<br>Pompilidae<br>Psychodidae<br>Psocoptera<br>Reduviidae<br>Staphylinidae<br>Tiphiidae<br>Vespidae	Araneae<br>Diptera<br>Coleoptera<br>Hemiptera<br>Heteroptera<br>Hymenoptera<br>Arachnid<br>Neoproptera
Sap suckers	Invertebrates that feed on the sugary sap produced in foliage and transported in the soft phloem tissue beneath the bark.	Aleyrodidae<br>Cicadas<br>Cicadelidae<br>Coreidae<br>Delphacidae<br>Flatidae<br>Geocoridae<br>Gerridae<br>Hemiptera<br>Hemerobidae<br>Lygaeidae<br>Miridae<br>Pentatomidae<br>Psyllidae<br>Rhyparochromidae<br>Scutelleridae<br>Terebrantia	Hemiptera<br>Heteroptera<br>Homoptera<br>Thysanoptera

		Tingidae	
Scavengers	Invertebrates that consume dead organisms.	Blattodae<br>Cucujidae<br>Hydrophilidae<br>Muscidae	Coleoptera<br>Diptera

Supplementary Table 2 Results for *lmer* models indicating chi-square (X^2) values of each of the model variables for individual shrub/tree and vegetation structure treatments ('tree only', 'shrub only' and 'tree and shrub').

	**df**	**Abundance**		**Richness**	
		X^2	***p* value**	X^2	***p* value**
Individual shrub and tree	1	19.83	0.01	12.50	0.001
Sampling periods	2	20.36	0.001	1.33	NS
Vegetation structure treatments	2	263.97	0.001	214.20	0.001
Sampling periods	2	24.78	0.001	36.79	0.001

Supplementary Table 3 Mean and standard error (in parenthesis) values of invertebrates sampled across plant forms at the level of individual plants and vegetation structure treatments at the plot scale in different sampling periods.

Sampling periods	**Plant forms (mean ± se)**		**Vegetation structure treatments (mean ± se)**		
	**Tree (n=32)**	**Shrub (n=128)**	**Tree only (n=16)**	**Shrub only (n=16)**	**Tree and shrub (n=16)**
Summer 2019-2020	42 (4.96)	78 (6.07)	39 (5.97)	320 (49.7)	347 (55.4)
Spring 2020	65 (19.1)	114 (11)	68 (13.8)	649 (47)	692 (62.6)
Summer 2020-2021	67 (9.05)	113 (8.30)	61(11.2)	470 (76.9)	500 (85.2)

Supplementary Table 4 Mean and standard error (in parenthesis) values for invertebrate abundance per plant and per plot for the respective functional groups.

**Functional groups**	**Individual shrub/tree**		**Vegetation structure (plot level)**		
	**Tree (n=32)**	**Shrub (n=128)**	**Tree only (n=16)**	**Shrub only (n=16)**	**Tree and shrub (n=16)**
**Detritivores**	6.97 (2.17)	10.8 (0.88)	5.72 (2.53)	43.87 (4.71)	46.4 (8.07)
**Herbivores**	34.0 (3.12)	54.7 (4.03)	20.5 (2.11)	156.5 (15.70)	149.2 (14.50)
**Parasitoids**	4.77 (0.44)	5.44 (3.44)	2.53 (0.25)	13.65 (1.23)	15.07 (1.35)
**Pollinators**	1.11 (0.28)	5.91 (1.01)	0.09 (0.03)	0.79 (0.34)	0.45 (0.08)
**Predators**	26.1 (3.04)	47.0 (2.72)	10.5 (1.42)	96.3 (9.36)	123.9 (13.60)
**Sap suckers**	3.55 (0.61)	4.48 (0.29)	4.37 (0.80)	31.3 (3.41)	27.4 (2.97)
**Scavengers**	0.4 (0.12)	1.07 (0.10)	0.18 (0.08)	2.65 (0.41)	2.91 (0.44)

Supplementary Table 5 Results of *lmer* models indicating chi-square (X^2) values of each of the model variables for invertebrate functional groups associated with individual tree and shrub. Plant type (tree or shrub) and sampling periods - summer 2019/20; spring 2020 and summer 2020/21, were specified as fixed effects and plot ID as a random effect.

**Functional groups**	**Variables**	**df**	X^2	***p* value**
Abundance of detritivores per individual plant	Individual shrub / tree	1	35.5	0.001
	Sampling periods	2	59.1	0.001
Abundance of herbivores per individual plant	Individual shrub / tree	1	8.27	0.001
	Sampling periods	2	60.6	0.001
Abundance of parasitoids per individual plant	Individual shrub / tree	1	2.36	ns
	Sampling periods	2	145.1	0.001
Abundance of pollinators per individual plant	Individual shrub / tree	1	231.7	0.001
	Sampling periods	2	1561.7	0.001
Abundance of predators per individual plant	Individual shrub / tree	1	30.5	0.001
	Sampling periods	2	124.9	0.001
Abundance of sap suckers per individual plant	Individual shrub / tree	1	4.29	0.03
	Sampling periods	2	7.20	0.02
Abundance of scavengers per individual plant	Individual shrub / tree	1	17.5	0.001
	Sampling periods	2	35.8	0.001

Supplementary Table 6 Results of *lmer* models indicating chi-square (X^2) values of each of the model variables for abundance of invertebrate functional groups per plot (response variable) for vegetation structure treatments. For all seven models, vegetation structure treatments and sampling periods - summer 2019/20; spring 2020 and summer 2020/21- were specified as fixed effects and plot ID as a random effect.

**Functional groups**	**Variables**	**df**	X^2	***p* value**
Abundance of detritivores per plot	Vegetation structure treatments	2	188.9	0.001
	Sampling periods	2	6.27	0.04
Abundance of herbivores per plot	Vegetation structure treatments	2	138.6	0.001
	Sampling periods	2	70.7	0.001
Abundance of parasitoids per plot	Vegetation structure treatments	2	86.1	0.001
	Sampling periods	2	15.2	0.001
Abundance of pollinators per plot	Vegetation structure treatments	2	15.1	0.001
	Sampling periods	2	6.96	0.03
Abundance of predators per plot	Vegetation structure treatments	2	277.4	0.001
	Sampling periods	2	6.05	0.04
Abundance of sap suckers per plot	Vegetation structure treatments	2	69.9	0.001
	Sampling periods	2	56.7	0.001
Abundance of scavengers per plot	Vegetation structure treatments	2	52.6	0.001
	Sampling periods	2	14.1	0.001

Supplementary Table 7 ANOVA results of *lmer* indicating chi-square (X^2) values of each of the model variables for abundance of invertebrate functional groups for combined datasets of individual shrub and tree, including significance for each explanatory variables namely canopy volume, LA (leaf area per plant) and presence of flowers. Plot ID and sampling periods were included as random effects in the models.

**Functional groups (Response variables)**	**Explanatory variables**	***df***	X^2	***p* value**
Abundance of detritivores per individual plant	Volume	1	4.90	0.02
	LA	1	21.5	0.001
	Presence of flowers	1	74.1	0.001
			$R^2m = 0.06$; $R^2c = 0.21$	
Abundance of pollinators per individual plant	Volume	1	90.7	ns
	LA	1	0.10	ns
	Presence of flowers	1	66.7	ns
			$R^2m = 0.14$; $R^2c = 0.20$	
Abundance of herbivores per individual plant	Volume	1	172.1	0.001
	LA	1	9.69	0.002
	Presence of flowers	1	104.7	0.001
			$R^2m = 0.19$; $R^2c = 0.66$	
Abundance of parasitoids per individual plant	Volume	1	80.2	0.001
	LA	1	15.2	0.001
	Presence of flowers	1	35.1	0.001
			$R^2m = 0.22$; $R^2c = 0.24$	
Abundance of predators per individual plant	Volume	1	53.3	0.001
	LA	1	106.9	0.001
	Presence of flowers	1	98.2	0.001
			$R^2m = 0.23$; $R^2c = 0.43$	
Abundance of sap suckers per individual plant	Volume	1	59.4	0.001
	LA	1	33.7	0.001
	Presence of flowers	1	59.2	0.001
			$R^2m = 0.17$; $R^2c = 0.53$	
Abundance of scavengers per individual plant	Volume	1	0.41	ns
	LA	1	94.7	0.001
	Presence of flowers	1	58.3	0.001
			$R^2m = 0.13$; $R^2c = 0.50$	

Supplementary Table 8 Results of *lmer* indicating chi-square (X^2) values of each of the seven model variables for abundance of invertebrate functional groups for vegetation structure treatments (plot level), including significance of explanatory variables (total volume, total LA (leaf area per plant) and no. of flowering plants) for each model. Plot ID and sampling periods were included as random effects in the models.

**Functional Group (Response variable)**	**Explanatory variable**	***df***	X^2	***p* value**
Abundance of detritivores	Total volume	1	0.25	ns
	Total LA	1	37.7	0.001
	No. of flowering plants	1	85.0	0.001
			$R^2c = 0.35$; $R^2m=0.41$	
Abundance of pollinators	Total volume	1	0.02	ns
	Total LA	1	10.6	0.001
	No. of flowering plants	1	10.6	0.001
			$R^2c = 0.13$; $R^2m=0.21$	
Abundance of herbivores	Total volume	1	15.9	0.01
	Total LA	1	0.00	ns
	No. of flowering plants	1	35.3	0.001
			$R^2c = 0.21$; $R^2m=0.30$	
Abundance of parasitoids	Total volume	1	14.3	0.001
	Total LA	1	9.49	0.001
	No. of flowering plants	1	22.3	0.002
			$R^2c = 0.20$; $R^2m=0.20$	
Abundance of predators	Total volume	1	7.77	0.001
	Total LA	1	2.13	ns
	No. of flowering plants	1	93.8	0.001
			$R^2c = 0.33$; $R^2m=0.33$	
Abundance of sap suckers	Total volume	1	12.6	0.001
	Total LA	1	4.18	0.04
	No. of flowering plants	1	77.6	0.001
			$R^2c = 0.35$; $R^2m=0.44$	
Abundance of scavengers	Total volume	1	6.47	0.01
	Total LA	1	8.14	0.001
	No. of flowering plants	1	45.2	0.001
			$R^2c = 0.22$; $R^2m=0.25$	
